# Squamous Cell Carcinoma of Left Buccal Alveolar Ridge

**DOI:** 10.7759/cureus.5271

**Published:** 2019-07-29

**Authors:** Pooja Patel, Hitanshu Dave, Rupak Desai, Louis-Marcel A Cesar, Priyank J Yagnik

**Affiliations:** 1 Rheumatology, Advocate Aurora Health, Brookfield, USA; 2 Internal Medicine, Hackensack Meridian Health - Jersey Shore University Medical Center, Neptune City, USA; 3 Cardiology, Atlanta Veterans Affairs Medical Center, Decatur, USA; 4 Emergency Medicine, St. Joseph's Medical Clinic, Waukesha, USA; 5 Pediatrics, University of Kansas School of Medicine, Wichita, USA

**Keywords:** alveolar ridge, gingival mass, buccal mucosa, squamous cell carcinoma, oral mass, hispanic

## Abstract

Squamous cell carcinoma (SCC) of the oral cavity accounts for 4% of malignancies in men and 2% of malignancies in women, and is responsible for 3% of all cancer deaths. Cancers of the gingiva often escape early detection and lead to a delay in intervention, since their signs and symptoms resemble common dental and periodontal infections. Here we present a case of a 55-year-old female patient who presented to our clinic with a left lower gingival mass for two weeks. Based on the clinical presentation, and possible differential diagnosis, this case highlights the importance of timely intervention and management.

## Introduction

Squamous Cell Carcinoma (SCC) accounts for the vast majority of malignancies of the oral cavity and oropharynx. There are more than half a million incident cases of squamous-cell carcinoma of the head and neck worldwide each year, primarily affecting the oropharynx, oral cavity, hypopharynx, and larynx. Nearly 50% of these patients die due to tumor-related complications. Only one-third of the patients are diagnosed during the early stage of the carcinoma. More than 50% of patients suffering from head and neck squamous cell carcinoma sustain local relapses and only up to 25% patients develop distant metastases. The prognosis of these patients is poor [1].

## Case presentation

A 55-year-old Hispanic female patient presented to the non-profit free medical health clinic with a chief complaint of left lower gingival mass for the past two weeks. It was associated with mild pain. The mass was friable, irregular and white. It had a cauliflower-like and rubbery consistency, and was gradually increasing in size. The patient did not report any fevers, chills, night sweats, or unintentional weight loss. The patient denied any recent dental or gingival trauma or infection, history of lifetime smoking or chewing tobacco, or similar symptoms in the past. The patient did not have a family history of tumor or carcinoma.

From the initial impression, the series of differential diagnosis consisted of 1) apical tooth infection-causing reactive granuloma formed over the adjacent gingiva 2) squamous cell carcinoma of buccal mucosa and, 3) benign hyperplasia of the gingiva. Since the clinic did not have an association with another non-profit dental clinic, various referral options were discussed with the patient, one of which included referral to a dental teaching institute. The patient did not have any health insurance and expressed reluctance to seek dental care outside of our non-profit free medical health clinic.

**Figure 1:**
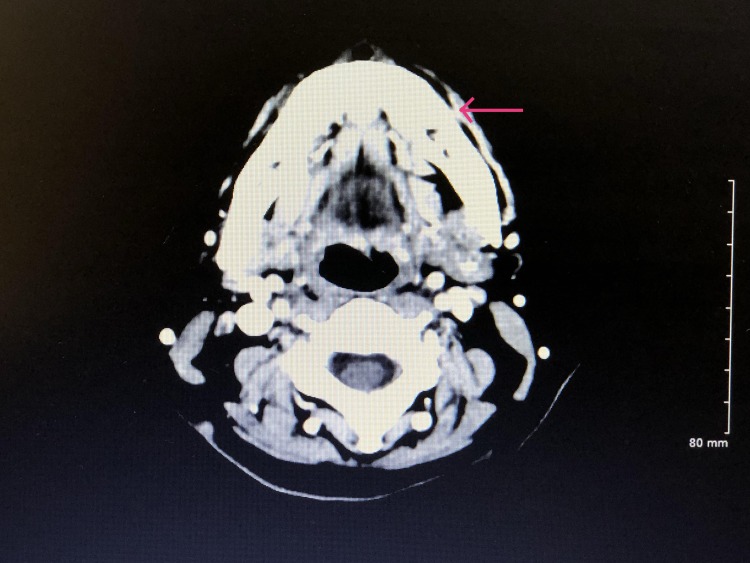
Computer Tomographic Angiography of Soft Tissue of Neck A soft tissue thickening with trace fluid and gas density (marked in red arrow) extending at least 2.5 cm x 0.6 cm thickness x 1.3 cm craniocaudal extent dimension lesion at the buccal aspect of the left mandibular alveolar ridge, presumably corresponding to a known biopsy-proved neoplasm. No osseous erosion or periosteal reaction of the underlying mandible is noted.

**Figure 2:**
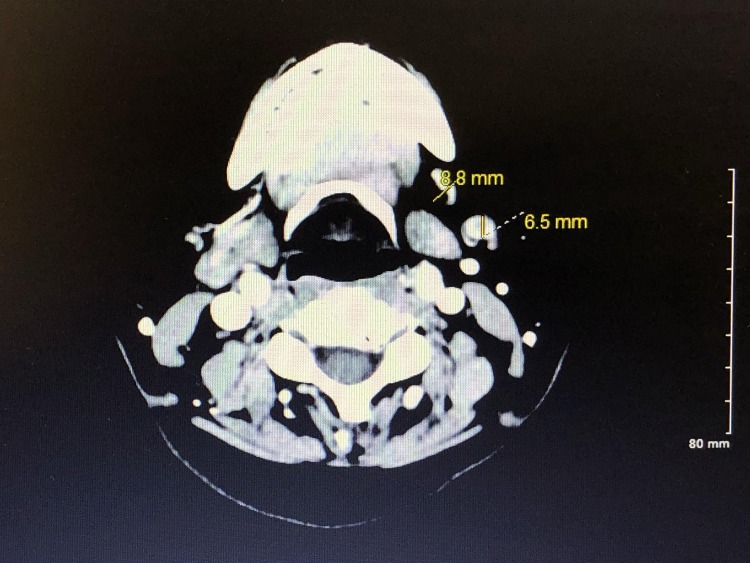
Computer Tomographic Angiography of Soft Tissue of Neck There is no evidence of pathologic lymphadenopathy; however, two left submandibular nodes, at level 1B (marked in yellow on the picture) show increased size (and one of which shows interval loss of central fatty hilum).

An otolaryngologist was contacted, who was willing to help at no cost to the patient. The patient consulted a surgeon, who recommended obtaining a biopsy of the mass for further evaluation. The biopsy showed squamous cell carcinoma of the alveolar ridge of the buccal mucosa. Following the biopsy results, the patient had a computerized tomography (CT) scan of the soft tissue neck with contrast to evaluate the extent of the malignancy (Figures 1, 2).

Due to concerns of possible malignancy on an imaging study, the patient underwent wide excision of the left posterior alveolar mass with rim mandibulectomy and tooth extractions, left selective neck dissection and buccal flap.

During her postoperative follow-up appointment with the surgeon, the patient was noted to have one lower molar remaining, with a well healing intra-oral surgical incision site. The patient had mild edema of the oral and buccal mucosa three weeks after the procedure. The neck incision healed with mild resolving post-operative edema. Overall, the patient has been recovering well and tolerated the procedure without any complications. 

During the entire course of treatment, the patient was educated and well informed about her condition, and the course of the treatment plan. She was well informed about the risks, side effects and prognosis of her condition. The patient was made aware of the clinic’s limitations due to limited resources. However, the patient was reassured, the clinic and the surgeon will work in conjugation with each other and will provide her the necessary care for her full recovery.

## Discussion

Oral carcinomas are amongst the most prevalent cancers in the world, and one of the leading causes of morbidity and mortality with squamous cell carcinoma (SCC) being the most common malignancy. Alveolar ridge SCC comprise 9% of all patients with oral SCC according to a report by Ildstad et al [2].

A retrospective review of 82 patients treated at the Massachusetts General Hospital from 1962 through 1976 for squamous cell carcinoma of the maxillary and mandibular alveolar ridge and soft and hard palates summarized the stage at first presentation, clinical features of the disease, analysis of current therapeutic modalities, survival statistics, and prevalence of second primary malignancies are analyzed and compared with reports from other large centers [2].

In parts of Southeast Asia, the incidence and prevalence of oral cancer are rising due to the increase in the use of tobacco, betel nuts, and hookah or lime to form a quid. The annual incidence rates of development of potentially malignant disorders were found to be 1.1-2.4/1000 in males and 0.2-1.3/1000 in females [3].

The most common site of intra-oral carcinoma is the tongue, followed by, in descending order, the floor of the mouth, palate, and gingiva (alveolar ridge). No site within the oral cavity is immune to SCC. However, there are certain locations where cancer occurs relatively frequently which are designated as “cancer-prone.” The lateral and ventral surfaces of the tongue and the floor of the mouth are the most common sites of oral SCC. This is based on the fact that the carcinogens within tobacco dissolve in the saliva and tend to pool and accumulate in the gravity-dependent regions of the oral cavity, also called the oral mucous reservoir [4].

When a patient presents with intr-aoral lesions, it is critical to obtain a detailed history and perform a thorough physical examination. Obtaining a history of nicotine/tobacco and alcohol use, dental history, trauma or injury is crucial. In the case reported, our differential diagnosis consisted of squamous cell carcinoma of the buccal mucosa, apical tooth infection causing reactive granuloma, and gingival benign hyperplasia. After obtaining a biopsy and Computer Tomography (CT) scan, a definitive diagnosis of squamous cell carcinoma of left alveolar buccal mucosa was made (Table 1).

**Table 1:** Approach to a patient with Squamous Cell Carcinoma N/A= Not applicable SCC= Squamous Cell Carcinoma

Patient management:	Specifications:
Comprehensive history	History of previous malignancies, including dermatologic malignancies. Further history of any symptoms of globus sensation, epistaxis, pain, otalgia, odynophagia, dysphagia, hemoptysis, or hoarseness.
Social history	History of use of tobacco; smoking or chewing
Physical examination	Thorough physical examination, especially detailed evaluation of the mass
Minimally invasive Fine Needle Aspiration of the neck mass	Specimen should be tested for viral etiology to help direct the search for the primary tumor. Most, but not all SCC can be linked to the presence of Epstein Barr virus or human papillomavirus
Office based nasopharyngolaryngoscopy	N/A
Imaging workup (magnetic resonance imaging, computed tomography, or positron emission tomography/ computed tomography with a high-resolution, contrast-enhanced diagnostic computed tomography component)	Note for the extent of the tumor and metastasis
If the primary tumor is not identified, panendoscopy with bilateral tonsillectomy and directed biopsies are indicated, and lingual tonsillectomy may be considered.	N/A
Management	Two main goals of treatment: Control of disease and Prevention of recurrence

Due to the lack of statistical data, it is easy to not consider squamous cell carcinoma among the Hispanic population, especially among female patients. With patients usually presenting with delayed presentation of their symptoms, patients often present with metastases. In this case, since our patient presented to the clinic within two weeks of onset of her symptoms, it enabled us to provide appropriate management to the patient’s carcinoma in a timely manner. CT scan did not show evidence of metastasis, therefore only limited surgical excision was required.

A retrospective cohort study showed that locoregional failure remained the most predominant pattern of failure for oral squamous cell carcinoma. Non-Caucasian race (hazard ratio [HR], 2.15; 95% confidence interval [CI], 1.22-3.81), T classification of the T3-T4 category (HR, 1.99; 95% CI, 1.18-3.35), and lymph node ratio greater than 10% (HR, 2.71; 95% CI, 1.39-5.27) were associated with poorer overall survival. Non-Caucasian patients also had a higher risk of locoregional failures (HR, 2.47; 95% CI, 1.28-4.79), whereas women were more likely to have distant metastasis (HR, 2.55; 95% CI, 1.14-5.71) [5].

## Conclusions

​The prognosis of squamous cell carcinoma is multifactorial and depends on size and location of the lesion, the extent of the lesion, presence or absence of metastasis, and the clinical staging of the disease. Survival of the patient is greatly dependent on early clinical detection and early intervention. In the present case, the patient presented to the non-profit health clinic during early onset of her symptoms, enabling the team of physicians to provide adequate medical attention to this patient who lacked medical insurance. We want to specially highlight the appropriate utilization of the available community resources in providing care to patients who are uninsured or underinsured.
